# Novel homozygous frameshift mutation of *ITGB3* in the Glanzmann thrombasthenia patient with abnormal bone metabolism and congenital bone defects

**DOI:** 10.1186/s13023-025-03700-9

**Published:** 2025-04-18

**Authors:** Yujiao Luo, Nina Guo, Yewei Wang, Ji Li

**Affiliations:** 1 Department of Hematology, Section of Hemostasis and Thrombosis, Institute of Molecular Hematology, The Second XiangYa Hospital, Central South University, No.139 Middle Renmin Road, Changsha, 410011 Hunan China; 2 National Clinical Research Center for Metabolic Diseases, Key Laboratory of Diabetes Immunology, Ministry of Education, Department of Metabolism and Endocrinology, The Second Xiangya Hospital of Central South University, Changsha, 410011 Hunan China; 3 Department of Hematology and Oncology, The Third Hospital of Changsha, Changsha, 410011 Hunan China

**Keywords:** Glanzmann thrombasthenia, Novel mutation, Bone defects, *ITGB3*, GPIIb/IIIa, Bone metabolism

## Abstract

**Background:**

Glanzmann thrombasthenia (GT) is a rare inherited bleeding disorder caused by dysfunction of the integrin αIIbβ3 in platelets. The subunit β3, encoded by *ITGB3* also plays a significant role in bone metabolism. Whether GT patients with β3 deficiency also suffer from bone pathology remains unclear.

**Method:**

The 21-year-old female patient presenting with bleeding diathesis and multiple congenital bone defects in her right hand, and her seven family members were included in the study. Whole exome sequencing as well as Sanger sequencing were conducted to identify GT-associated mutations within the family. The platelet function of the family was detected by the platelet aggregation test and thromboelastography (TEG). The expression levels of CD41 (αIIb) and CD61 (β3) on the platelet surface and total in platelet were detected by flow cytometry and Western blot. Bioinformatics analysis was used to evaluate the pathogenicity of mutation sites and their effects on protein structure and function. X-ray imaging, bone densitometry and bone metabolism index were performed to evaluate bone development and metabolism.

**Result:**

A novel homozygous frameshift mutation c.2143_2158delinsCT (p.Lys715Leufs*36) of *ITGB3* was found in the proband. Platelet aggregation by ADP, collagen, epinephrine, and arachidonic acid was absent, TEG showed hypocoagulability and decreased platelet function, and the expression levels of αIIb and β3 on the platelet surface and total in platelet were significantly reduced (< 5%) in the proband. The parents, second elder sister and grandmother of proband were heterozygous carriers without bleeding symptoms and had normal platelet aggregation function and αIIb/β3 protein expression. Structural modeling strongly suggested that the mutation creates a truncation in cytoplasmic domains of β3, resulting in the mutant β3/αIIbβ3 inactivated and low expression. The proband was born with partial absence of phalanges in digits 2–4 and the deformity of fingers 1 and 5 in her right hand, bone densitometry indicated significant osteopenia and increased risk of fracture in her right radius, and no other gene mutations related to bone pathology were identified.

**Conclusion:**

A novel mutation of *ITGB3* which results in GT was identified. This is the third reported case of GT combined with bone defect. Our work expands *ITGB3* mutation spectrum and provide further insights into the potential association between GT and bone development and metabolism.

## Introduction

Glanzmann thrombasthenia (GT) is a rare autosomal recessive inherited bleeding disorder. It is characterized by a quantitative or qualitative defect in integrin αIIbβ3, also referred to as glycoprotein GPIIb/IIIa, which is crucial for platelet aggregation and normal hemostasis. The genes *ITGA2B* and *ITGB3* encode subunits αIIb (CD41) and β3 (CD61) respectively. αIIb is predominantly confined to cells of the megakaryocyte lineage. In contrast, β3 is widely present in various cell types in the form of integrin aVβ3 and plays a significant role in bone metabolism. The deficiency of β3 not only causes bleeding but may also impact bone metabolism. However, the evidence obtained from β3-deficient mice is inconsistence with the data derived from patients with *ITGB3* defects. Whether GT patients with β3 deficiency also suffer from bone pathology remains unclear, and only a few cases of GT patient with bone defects have been reported. In this study, a novel homozygous frameshift mutation of *ITGB3* was identified in a young female GT patient presenting with bleeding diathesis, osteopenia, and congenital bone defects. To the best of our knowledge, this is the third case reporting a GT patient with bone defects. This finding expands *ITGB3* mutation spectrum and will contribute more information regarding the association between GT and bone development and metabolism.

## Materials and methods

### Subjects

**Fig. 1:**
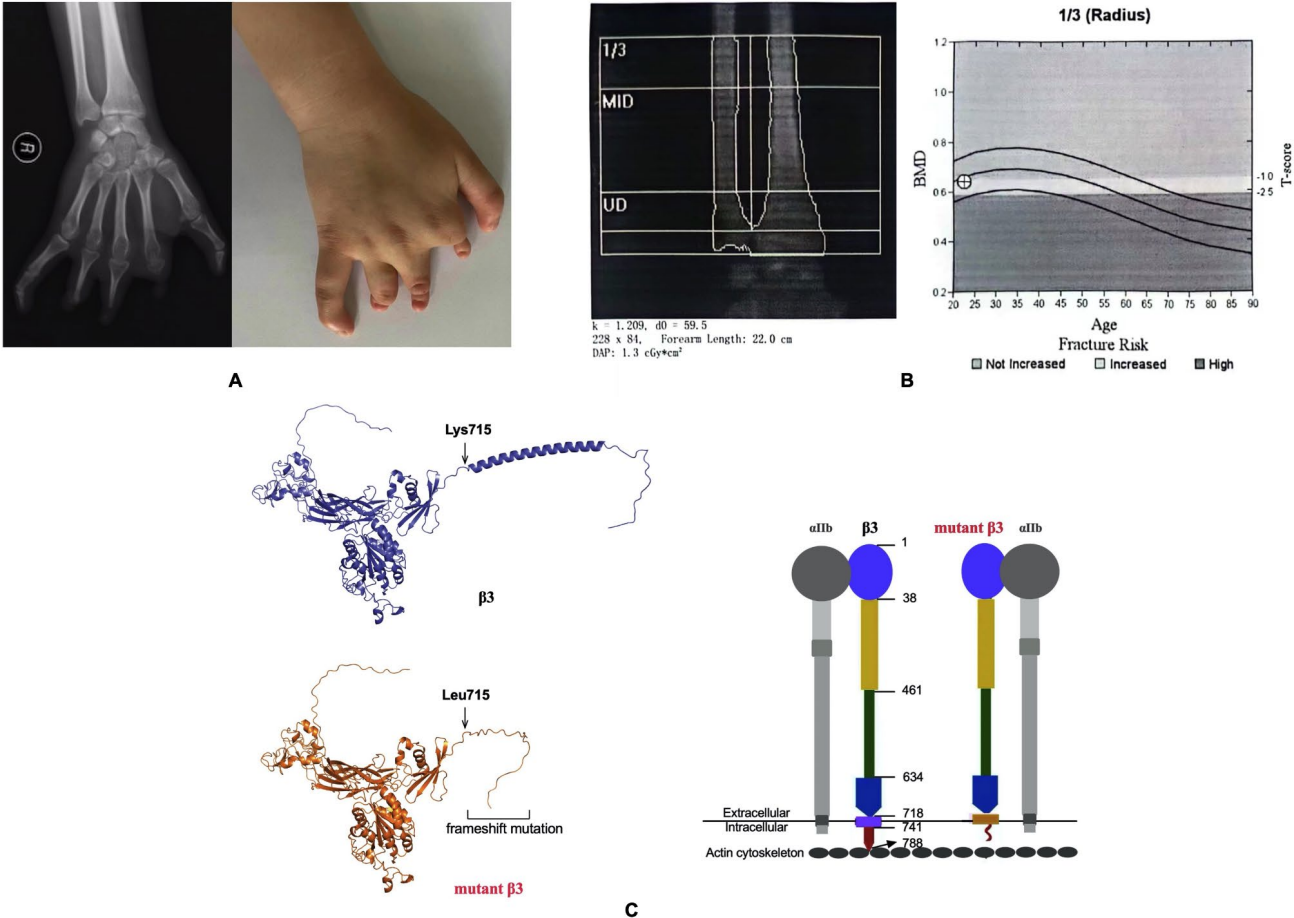
X-ray imaging, BMD of the proband, and molecular modeling of the mutant β3. (**A**) X-ray imagine of the proband’s right hand. The X - ray image of the proband’s right hand reveals that the proband was born with partial absence of the phalanges in digits 2–4, as well as deformities in digits 1 and 5. (**B**) Radial BMD Scan of the Proband’s Right Forearm. Schematic representation of the radial BMD scan of the right forearm of the proband indicated a significantly lower radial BMD, suggesting significant osteopenia and increased risk of fracture. (**C**) Molecular modeling analysis of the mutant β3. The mutation results in the substitution of lysine with leucine at the 715th amino acid position of β3, followed by premature termination at the 750th amino acid. This truncation leads to the loss of the cytoplasmic domains (residues 742–788), ultimately causing inactivation and reduced expression of the β3/αIIbβ3

The proband/patient is a 21-year-old unmarried and nulliparous Chinese female presenting with recurrent skin petechias, epistaxis, gingival bleeding, and menorrhagia. She had been hospitalized on two occasions due to bleeding. In addition to the hemorrhagic diathesis, the proband was born with multiple bone defects in her right hand (Fig. 1-A). Two sisters of her mother passed away at a young age, one at 7 years and other at 20 days old. Among her living family members, except for the proband, none exhibited bleeding symptoms. The proband and seven family members were enrolled in the study. The research involving human participants was reviewed and approved by the Medical Ethics Committee of The Second XiangYa Hospital of Central South University.

### Sample collection

Peripheral blood samples were collected from each member of the pedigree and healthy controls subjects. Citrated samples (3.2% trisodium citrate at 1:9 ratio) were used for coagulation tests, platelet aggregation studies and thromboelastography (TEG) analyses. Platelet count determination, assessment of the expression of CD41 and CD61, Western blotting, and DNA extraction were carried out on EDTA-anticoagulated samples. Bone metabolism indices were measured in serum sample.

### Platelet-related and coagulation function examination

Peripheral blood smear was performed to observe the morphology of platelets. Platelet count was performed in Sysmex-XN20 automated analyzer (Sysmex, Japan). prothrombin time (PT), activated partial thromboplastin time (APTT), fibrinogen level and Thrombin Time (TT) were measured using the ACL-TOP 700 automatic coagulometer (HemosIL™, IL, MA, USA) to evaluate the coagulation profile. Kaolin activated thromboelastography (TEG) was performed on Haema TA (Medcaptain, China) within 1 h of sample collection using citrated whole blood. The test was conducted until all parameters, including R-time (R), K-time (K), Angle and Maximum amplitude (MA), were obtained. Platelet aggregation tests were performed using the model 700 aggregometer (Chronolog corporation, USA) with agonists such as adenosine diphosphate (ADP, 2.5mM), collagen (Col,1 µg/ml), epinephrine (EPI, 5mM) and arachidonic acid (AA, 0.25 mM) (Helena Laboratories, USA).

### Detection of the expression of ΑIIb and Β3

The expression of CD41 and CD61 on the platelet surface was detected by flow cytometry (FACSCantoTMII, BD, USA). All the primary antibodies (BD, USA) were directly labeled with fluorescein isothiocyanate (FITC), obviating the need for a secondary antibody. The mean fluorescence intensity was analyzed in the forward scatter (FSC) and side scatter (SSC) logarithmic model, and the data were analyzed by FlowJo v10.8.1 software.

The expressions of total αIIb and β3 in platelets were analyzed by Western blotting. Peripheral blood was anticoagulated with EDTA and centrifuged at 200×g for 6 min to obtain platelet-rich plasma (PRP). The PRP was transferred to a new centrifuge tube and centrifuged at 1500×g for 10 min, the supernatant was discarded, and the platelets were resuspended in phosphate-buffered saline (PBS) and centrifuged again at 1500×g for 5 min. Subsequently, the supernatant was discarded, and an appropriate amount of lysate was added to extract total platelet protein for Western blotting. The primary antibody used in Western blotting included rabbit monoclonal antibody against CD41, integrin β3 rabbit polyclonal antibody, and β-actin antibody (Abcam, Cambridge, MA, USA). HRP-conjugated Goat anti-rabbit IgG was used as the secondary antibody.

### Whole exome sequencing

Genomic DNA was extracted from peripheral blood samples of all subjects. Whole exome sequencing (WES) was performed for the proband at Kindstar Global Company (Wuhan, China). Variants were annotated and filtered according to the guidelines issued by the American College of Medical Genetics and Genomics (ACMG). GT Database. (http://sinaicentral.mssm.edu/intranet/research/glanzmann/menu), EXAC Database (http://exac.broadinstitute.org/terms), Ensembl Database (http://asia.ensembl.org/index.html), gnomAD (http://www.gnomad-sg.org), OMIM (http://omim.org/), PubMed (http://www.ncbi.nlm.nih.gov/pubmed), ClinVar.

(https://www.ncbi.nlm.nih.gov/clinvar/), HGMD (http://www.hgmd.cf.ac.uk/ac/index.php) and other database were used to assess the clinical significance of identified mutations.

### Polymerase chain reaction

Sanger sequencing was carried out to confirm the variant of *ITGB3* gene in the family. Polymerase chain reaction (PCR) primers were designed as follows: forward primer (5′- CCACTCAGTGCAGATTATTGCTGTCC-3′) and reverse primer (5′-GCCCAGCCTGGAAGTAACCA-3′). The DNA was amplified using the following procedure: 95 °C for 5 min; 40 cycles at 95 °C for 30 s, 60 °C for 30 s, 72 °C for 30 s; 72 °C for 5 min. Sequencing was performed by an ABI 3130 DNA analyzer.

### Bioinformatics analysis

The pathogenicity of the mutation was evaluated using the Protein Variation Severity Score 1 (PVS1) and Polymorphism Metrics 2 (PM2) [1, 2]. Homology modeling of the wild-type and mutant β3 protein structures was performed using SWISS-MODEL (https://swissmodel.expasy.org/), and the three-dimensional (3D) structures were visualized and analyzed using Pymol v2.6 software.

### Bone development and metabolism

The average coverage of the target regions in WES was 124.43X, with 98.96% of the target regions covered at least 10x. Copy number variation (CNV) analysis was performed. Databases such as PubMed, gnomAD, OMIM, ClinVar, EXAC, Ensembl, and HGMD were thoroughly searched to identify gene mutations associated with bone pathology. X-ray imaging and bone mineral density (BMD) tests using dual-energy X-ray absorptiometry (DXA) were conducted for the proband. Given the strong correlation between bone metabolism indices and age, the bone metabolism study was restricted to the proband and her sisters (the eldest sister without the mutation and the second older sister who is a heterozygous carrier). BMD tests were also performed for the proband’s sisters. Osteocalcin, total Procollagen Type 1 N-Terminal Propeptide (P1NP), β-CrossLaps, 25-hydroxyvitamin D, parathyroid hormone, alkaline phosphatase, blood calcium, and blood phosphorus levels of the proband and her sisters were measured using a fully automated biochemical analyzer (Roche, Switzerland).

## Results

### Identification of Glanzmann thrombasthenia

**Fig. 2:**
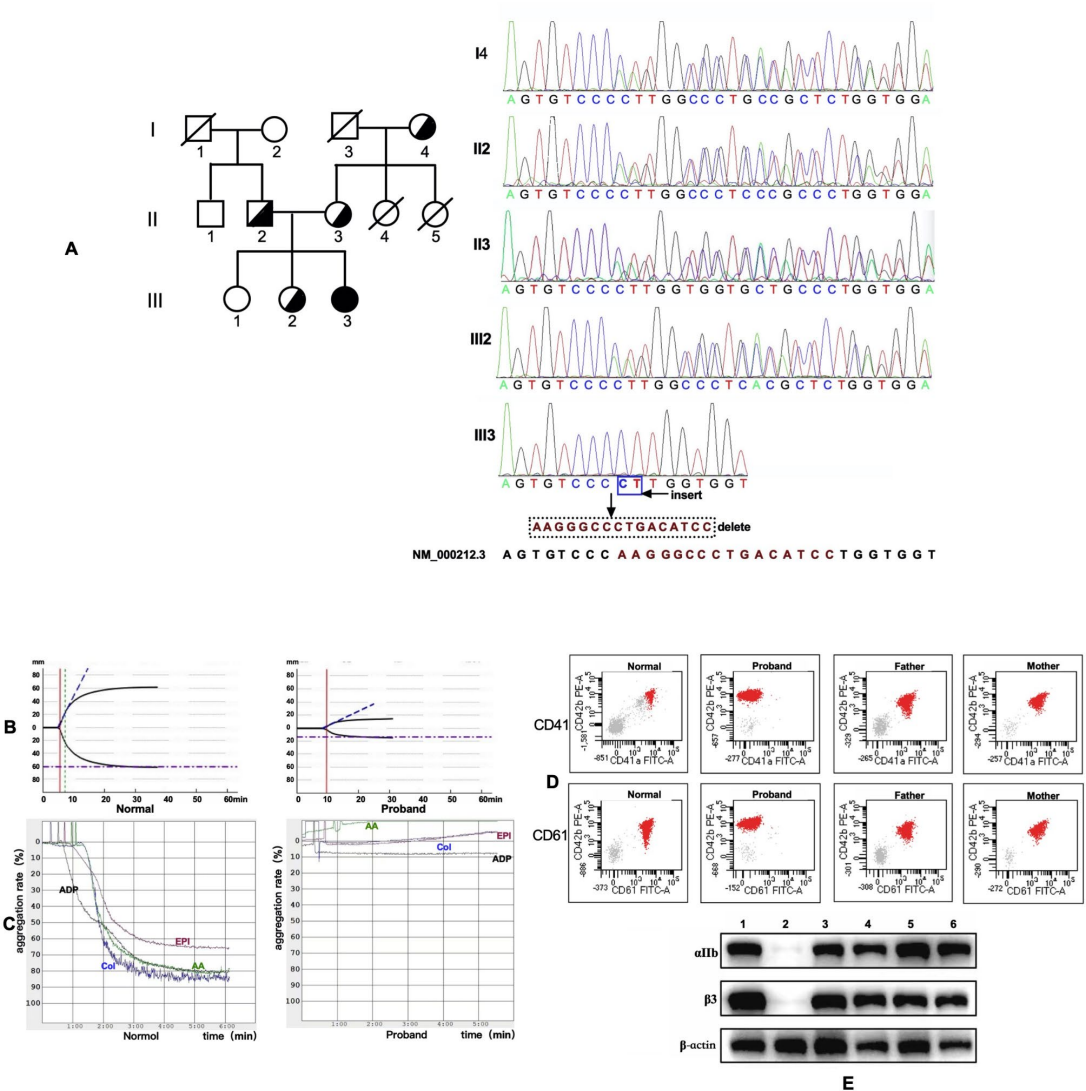
Family Pedigree, sanger sequencing, platelet aggregation curves, thrombelastogram and expression of αIIb and β3. (**A**) Pedigree of the GT family and sanger sequencing validation. NM_000212.3 is the reference transcript. III3 (proband) had a homozygous frameshift mutation c.2143_2158delinsCT (16 nucleotides were deleted, and CT were inserted in their place) in exon 14 of *ITGB3* gene. I4 (grandmother), II2 (father), II3 (mother), abd III2 (the second older sister) were found to be heterozygous carriers. II4 and II5 passed away at the ages of 7 years and 20 days, respectively. (**B**) Platelet aggregation in the proband. Platelet aggregation by ADP, Col, EPI, and AA was absent in the proband. (**C**) TEG analysis. TEG results indicated hypocoagulability, as well as reduced platelet function and fibrinogen activity. (**D**) Expression of CD41 and CD61 on platelet surface. The expression of CD41 and CD61 on the platelet surface in the proband was significantly reduced (< 5%). In contrast, the expression levels were normal in the heterozygous carriers (for simplcity, only the parents of the proband are presented in the figure). (**E**) Expressions of total αIIb and β3 in platelets. The expressions of total αIIb and β3 in platelets of the proband were significantly reduced (< 5%) but were normal in the heterozygous carriers. 1: normal control, 2: proband, 3–6: the parents, second older sister, and grandmother of proband, respectively

The counts and morphology of platelet, APTT, PT, fibrinogen levels, and TT were within normal ranges for all family members. TEG, platelet aggregation, and the expression levels of CD41 and CD61 of pedigree members were also normal in all pedigree members except the proband. Proband findings: TEG revealed hypocoagulability and decreased platelet function (MA = 13.2, Reference range: 50–70) and fibrinogen activity (Angle = 27.8°, reference range: 53°-72°) (Fig. 2-B). Platelet aggregation in response to ADP, Col, EPI, and AA was absent (Fig. 2-C). The expression levels of αIIb and β3 on the platelet surface and total in platelet were significantly reduced (< 5%) (Fig. 2-D, E). Based on these findings, the proband was diagnosed with type I GT.

### Identification of a novel frameshift mutation of *ITGB3* and family pedigree investigation

WES identified a homozygous frameshift mutation in exon 14 of *ITGB3* in the proband: NM_000212.3: c.2143_2158delinsCT (p.Lys715Leufs*36). This mutation was confirmed by Sanger sequencing. The parents, second older sister, and grandmother of the proband were found to be heterozygous carriers, consistent with the autosomal recessive inheritance pattern of GT (Fig. 2-A). This mutation has not been previously reported in any database or literature.

### Prediction of the variant C.2143_2158 delins CT

According to the ACMG guidelines, the frameshift variant *ITGB3* NM_000212.3: c.2143_2158delinsCT (p. Lys715 Leufs*36) was classified as ‘‘likely pathogenic” based on the criteria PVS1 and PM2. The 3D structural model of the β3 subunit demonstrated that the variant results in 16 nucleotides (c.2143_2158del) were deleted and replaced with “CT”, altering the 715th amino acid from lysine to leucine and introducing a premature stop codon at the 750th amino acid (normal protein length: 788 amino acids). This truncation leads to the loss of cytoplasmic domains, mRNA degradation, and subsequent inactivation and low expression of the β3/αIIbβ3 complex (Fig. 1-C).

### Bone development and metabolism

No genetic mutations associated with bone pathologies were identified through WES. X-ray imaging revealed congenital absence of phalanges in digits 2–4 and deformities in digits 1 and 5 of the proband’s right hand (Fig. 1-A). No bone defects or deformities were observed in other family members. DXA indicated significantly lower radial BMD in the proband’s right forearm, meeting the diagnostic criteria for osteopenia and suggesting an increased risk of fracture (Fig. 1-B). The proband’s sisters exhibited normal BMD and bone metabolism indices except for varying degrees of decreased 25-hydroxyvitamin D (eldest sister: 58 nmol/L; second older sister nmol/L: 47 nmol/L; the proband: 29 nmol/L; reference range: 75–250 nmol/L). The 25-hydroxyvitamin D level in the proband was the lowest. Moreover, the proband exhibited elevated parathyroid hormone levels (81.5 U/L; reference range: 18.4–80.1 U/L) and decreased alkaline phosphatase levels (71.0 U/L; reference range: 81.0–454.0 U/L). These findings indicated certain abnormalities in bone metabolism, which were consistent with the osteopenia detected by the BMD examination.

## Discussion

In the study, we identified a novel homozygous frameshift mutation NM_000212.3: c.2143_2158delinsCT (p.Lys715Leufs*36), in the *ITGB3* gene, which result in GT. The mutation introduced a premature stop codon, leading to the truncation of β3. Consequently, the expression and function of αIIbβ3 were severely compromised, resulting in the absence of platelet aggregation and bleeding diathesis. The mutation has not been previously reported in any database or literature. Thus, our findings not only expand the diagnostic spectrum of GT but also provide valuable insights and information for the genetic counseling.

Notably, we also discovered abnormal bone metabolism in the GT proband and reported the third case of a GT patient with bone defects. This contributes to a better understanding of the association between bone pathology and GT caused by β3 deficiency. Integrin αIIbβ3 is synthesized individually in the megakaryocytes followed by the complex formation in the endoplasmic reticulum. Incorrect folding or failure forming of the complex leads to rapid intracellular degradation. Therefore, a genetic defect in either αIIb or β3 can cause a deficiency in the membrane expression of the entire integrin αIIbβ3 complex. We propose that the proband lacks the αIIbβ3 complex due to the frameshift mutation, which likely leads to premature termination of the protein, most probably through nonsense - mediated decay at the mRNA level. Since the heterozygous mutation does not cause base deletion and protein truncation, the heterozygous carriers in the GT family exhibited normal platelet aggregation function and αIIb/β3 protein expression and did not show any bleeding symptoms.

Molecular modelling strongly suggests that the mutation creates a truncation in cytoplasmic domains of β3, rendering the mutant β3/αIIbβ3 inactivated and causing low expression. The cytoplasmic domain is involved in the phosphorylation of multiple tyrosines and threonine residues, mediating series of of critical interactions. For example, it ensures the binding of talin and kindin-3, which are essential for αIIbβ3 activation, regulates cytoskeletal dynamics, responds to growth factor signals, influences transcriptional regulation, and determines integrin localization and surface expression [3]. We speculate that the mutant β3 lacking cytoplasmic domains may also have an impact on bone physiology.

β3 exists in osteocytes in the form of aVβ3 and plays an important role in bone metabolism [4–12]. Most patients with disease-causing mutations of *ITGB3* also lack αvβ3. However, studies on the altered bone pathophysiology in condition of β3 deficiency have produced contradictory results. Some studies have demonstrated a critical role of integrin β3 subunits in osteoclast migration and maturation [9, 10], but the specific effects of β3 deficiency on bone pathophysiology remain unclear. Hodivala-Dilke KM found that, in addition to bleeding symptoms, the β3-deficient GT mouse model also exhibited osteosclerosis with age. β3-null mice possessed increased numbers of osteoclasts which were non-functional and unable to resorb bone [13]. Significantly, the Ser752Pro mutation also impaired osteoclast function when incorporated into the murine β3 cytoplasmic tail. Moreover, the peptidomimetic antagonist of the aVβ3 integrin inhibited bone resorption in vitro and postmenopausal osteoporosis in vivo. The loss of β3-integrin in myeloma plasma cells impacted the bone-resorbing function and prevented the formation of erosion pits [14]. However, bone mass thickening has not been observed in GT patients, while GT combined with congenital osteoporosis has been reported [15]. Osteoporosis has also been found in mice with myeloid β3 deficiency [16]. In our study, the young proband showed abnormal bone metabolism and osteopenia, which is inconsistent with her age.

To date, only 2 cases of GT patient with bone defects have been reported [8, 17]. Grimaldi et al. [17] described destructive changes in both ankle joints, with total loss of the tibia talar joint in a GT patient and attributed this to remote hemorrhage. Nurden et al. [8] found that a GT patient with a congenital bone defect had two mutations, Arg327His and Gly381Arg, on αIIb. These complex mutations affected the interface of αIIb and β3 subunits and destabilize the β3 propeller by pushing back the surrounding residues. We report the third case, in which the GT patient with congenital bone defect had a novel frameshift mutation on β3. This mutation led to premature termination and the loss of the cytoplasmic domains of β3. Although cases of the co-existence of bone defects and GT are scarce, there is evidence of platelet-associated bone defects, such as Thrombocytopenia-absent radius (TAR) syndrome which is characterized by bilateral absence of radius with the presence of both thumbs and thrombocytopenia. This indicates that platelet dysfunction is closely related to bone defects.

Previous studies have focused predominantly on osteoclasts, which may be one reason for the inconsistent results. Recently, it has been found that the loss of β3 integrin in osteocyte did not affect osteoclast formation, but significantly reduced the osteoblast-mediated bone formation rate and the osteogenic differentiation of the bone marrow stromal cells in the bone microenvironment [18, 19]. The development of bone cortex and femur length was impaired after silencing integrin β3 [20]. DEL-1 can activate β3 integrin-FAK-ERK1/2-RUNX2 pathway in osteoprogenitors and promote new bone formation in mice [21]. Moreover, recently, a new application of integrins has emerged, namely in biomaterials for bone repair in cases of non-healing bone defects caused by osteoarthritis, traumatic injury, and cancer [22]. The novel mutation may also affect bone formation, resulting in the skeletal defects of the proband, which warrants further study. Additionally, WES primarily targets exonic regions and cannot detect variants in non-coding regions, such as regulatory sequences and introns, which may play significant roles in disease. Variant interpretation is also limited to current knowledge and databases. So far, whether the bone defects are related to the GT gene mutation or are merely a coincidence remains unclear and requires further confirmation through the accumulation of more clinical cases and in vivo experiments. The proband is a young woman of childbearing age, and there are many variables in her future. We will continue to follow up and study the association between bone pathology and GT caused by β3 dificiency and provide fertility risk prevention and medical assistance for her.

## Conclusion

We identified a novel mutation of *ITGB3* in a GT patient with abnormal bone metabolism and congenital bone defects. Our work expands *ITGB3* mutation spectrum and and provides more information on the association of GT with bone development and metabolism.

## Data Availability

All data generated or analyzed during this study are included in this published article.

## References

[1] Abou Tayoun AN, Pesaran T, DiStefano MT, Oza A, Rehm HL, Biesecker LG, Harrison SM. ClinGen sequence variant interpretation working G. Recommendations for interpreting the loss of function PVS1 ACMG/AMP variant criterion. Hum Mutat. 2018;39:1517–24. Epub 2018/09/08. 30192042 10.1002/humu.23626 PMC6185798

[2] Richards S, Aziz N, Bale S, Bick D, Das S, Gastier-Foster J, Grody WW, Hegde M, Lyon E, Spector E, et al. Standards and guidelines for the interpretation of sequence variants: a joint consensus recommendation of the American college of medical genetics and genomics and the association for molecular pathology. Genet Med. 2015;17:405–24. Epub 2015/03/06. 25741868 10.1038/gim.2015.30 PMC4544753

[3] Aretz J, Aziz M, Strohmeyer N, Sattler M, Fassler R. Talin and kindlin use integrin tail allostery and direct binding to activate integrins. Nat Struct Mol Biol. 2023;30:1913–24. Epub 2023/12/13. 38087085 10.1038/s41594-023-01139-9 PMC10716038

[4] Chen S, He T, Zhong Y, Chen M, Yao Q, Chen D, Shao Z, Xiao G. Roles of focal adhesion proteins in skeleton and diseases. Acta Pharm Sin B. 2023;13:998–1013. Epub 2023/03/28. 36970189 10.1016/j.apsb.2022.09.020 PMC10031257

[5] Aso K, Soutome T, Satoh M, Aoki T, Ogura H, Yamamoto T, Kanno H, Takahashi H. Association of autosomal-recessive-type distal renal tubular acidosis and Glanzmann thrombasthenia as a consequence of runs of homozygosity. Clin Case Rep. 2022;10:e6070. Epub 2022/07/23. 35865781 10.1002/ccr3.6070 PMC9295683

[6] Botero JP, Lee K, Branchford BR, Bray PF, Freson K, Lambert MP, Luo M, Mohan S, Ross JE, Bergmeier W, et al. Glanzmann thrombasthenia: genetic basis and clinical correlates. Haematologica. 2020;105:888–94. Epub 2020/03/07. 32139434 10.3324/haematol.2018.214239 PMC7109743

[7] Nurden AT. Should studies on Glanzmann thrombasthenia not be telling Us more about cardiovascular disease and other major illnesses? Blood Rev. 2017;31:287–99. Epub 2017/04/12. 28395882 10.1016/j.blre.2017.03.005

[8] Nurden AT, Fiore M, Nurden P, Heilig R, Pillois X. Are bone defects in rare patients with Glanzmann’s thrombasthenia associated with ITGB3 or ITGA2B mutations? Platelets. 2011;22:547–51. Epub 2011/05/12. 21557682 10.3109/09537104.2011.573600

[9] Yu D, Li Z, Cao J, Wei G, Shen F. LSD1 knockdown confers protection against osteoclast formation by reducing histone 3 lysine 9 monomethylation and dimethylation in ITGB3 promoter. Acta Histochem. 2023;125:152073. Epub 2023/07/09. 37422927 10.1016/j.acthis.2023.152073

[10] Rashid S, Wilson SG, Zhu K, Walsh JP, Xu J, Mullin BH. Identification of differentially expressed genes and molecular pathways involved in osteoclastogenesis using RNA-seq. Genes (Basel) 2023;14. Epub 2023/04/28. 10.3390/genes14040916 PMC10137460 37107674

[11] Yu D, Li Z, Cao J, Shen F, Wei G. microRNA-25-3p suppresses osteogenic differentiation of BMSCs in patients with osteoporosis by targeting ITGB3. Acta Histochem. 2022;124:151926. Epub 2022/07/02. 35777302 10.1016/j.acthis.2022.151926

[12] Yang L, Chen H, Yang C, Hu Z, Jiang Z, Meng S, Liu R, Huang L, Yang K. Research progress on the regulatory mechanism of integrin-mediated mechanical stress in cells involved in bone metabolism. J Cell Mol Med. 2024;28:e18183. Epub 2024/03/20. 38506078 10.1111/jcmm.18183 PMC10951882

[13] Hodivala-Dilke KM, McHugh KP, Tsakiris DA, Rayburn H, Crowley D, Ullman-Cullere M, Ross FP, Coller BS, Teitelbaum S, Hynes RO. Beta3-integrin-deficient mice are a model for Glanzmann thrombasthenia showing placental defects and reduced survival. J Clin Invest. 1999;103:229–38. Epub 1999/01/23. 9916135 10.1172/JCI5487 PMC407888

[14] Tucci M, De Palma R, Lombardi L, Rodolico G, Berrino L, Dammacco F, Silvestris F. beta(3) integrin subunit mediates the bone-resorbing function exerted by cultured myeloma plasma cells. Cancer Res. 2009;69:6738–46. Epub 2009/08/06. 19654300 10.1158/0008-5472.CAN-09-0949

[15] Yarali N, Fisgin T, Duru F, Kara A. Osteopetrosis and Glanzmann’s thrombasthenia in a child. Ann Hematol. 2003;82:254–6. Epub 2003/04/23. 12707732 10.1007/s00277-002-0571-3

[16] Morgan EA, Schneider JG, Baroni TE, Uluckan O, Heller E, Hurchla MA, Deng H, Floyd D, Berdy A, Prior JL, et al. Dissection of platelet and myeloid cell defects by conditional targeting of the beta3-integrin subunit. FASEB J. 2010;24:1117–27. Epub 2009/11/26. 19933310 10.1096/fj.09-138420 PMC2845430

[17] Grimaldi CM, Chen F, Wu C, Weiss HJ, Coller BS, French DL. Glycoprotein IIb Leu214Pro mutation produces Glanzmann thrombasthenia with both quantitative and qualitative abnormalities in GPIIb/IIIa. Blood. 1998;91:1562–71. Epub 1998/03/21. 9473221

[18] Qin L, Chen Z, Yang D, He T, Xu Z, Zhang P, Chen D, Yi W, Xiao G. Osteocyte beta3 integrin promotes bone mass accrual and force-induced bone formation in mice. J Orthop Translat. 2023;40:58–71. Epub 2023/07/17. 37457310 10.1016/j.jot.2023.05.001 PMC10338905

[19] Zou Z, Liu R, Wang Y, Xing Y, Shi Z, Wang K, Dong D. IL1RN promotes osteoblastic differentiation via interacting with ITGB3 in osteoporosis. Acta Biochim Biophys Sin (Shanghai). 2021;53:294–303. Epub 2021/01/26. 33493267 10.1093/abbs/gmaa174

[20] Geng X, Tang Y, Gu C, Zeng J, Zhao Y, Zhou Q, Jia L, Zhou S, Chen X. Integrin alphaVbeta3 antagonist-c(RGDyk) peptide attenuates the progression of ossification of the posterior longitudinal ligament by inhibiting osteogenesis and angiogenesis. Mol Med. 2024;30:57. Epub 2024/05/03. 38698308 10.1186/s10020-024-00822-x PMC11067224

[21] Yuh DY, Maekawa T, Li X, Kajikawa T, Bdeir K, Chavakis T, Hajishengallis G. The secreted protein DEL-1 activates a beta3 integrin-FAK-ERK1/2-RUNX2 pathway and promotes osteogenic differentiation and bone regeneration. J Biol Chem. 2020;295:7261–73. Epub 2020/04/14. 32280065 10.1074/jbc.RA120.013024 PMC7247308

[22] Tollabi M, Poursalehi Z, Mehrafshar P, Bakhtiari R, Hosseinpour Sarmadi V, Tayebi L, Haramshahi SMA. Insight into the role of integrins and integrins-targeting biomaterials in bone regeneration. Connect Tissue Res. 2024;65:343–63. Epub 2024/09/22 20:51. 39297793 10.1080/03008207.2024.2396002 PMC11541888

